# Supplementary figures and images for: Predicting Parkinson’s disease trajectory using clinical and functional MRI features: A reproduction and replication study
Source: PLoS One. 2025 Feb 21;20(2):e0317566. doi: 10.1371/journal.pone.0317566 (PMC11844873; doi:10.1371/journal.pone.0317566)

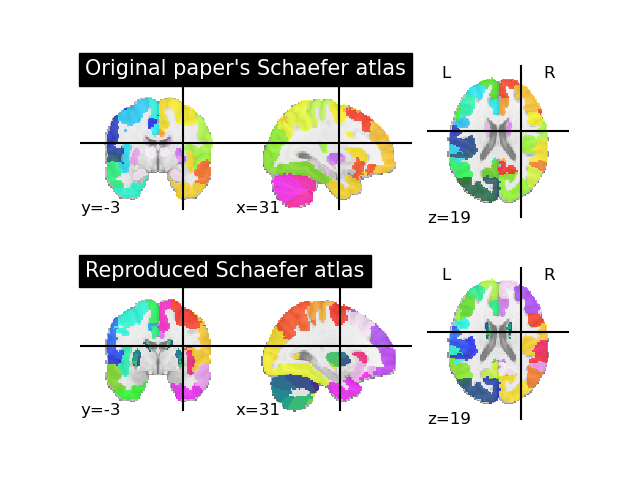

Supplement: S1 Fig — (TIF) [file pone.0317566.s001.tif]
