# Supplementary material for: Predicting Parkinson’s disease trajectory using clinical and functional MRI features: A reproduction and replication study
Source: PLoS One. 2025 Feb 21;20(2):e0317566. doi: 10.1371/journal.pone.0317566 (PMC11844873; doi:10.1371/journal.pone.0317566)
Supplement: S1 Table — (PDF) [file pone.0317566.s002.pdf]

Study file	Feature	Columns	Encoding
Demographics	Race	“RAWHITE”, ”HISPLAT”, “RAINDALS”, ”RABLACK”, “RAASIAN”, “RAHAWOPI”, “RANOS”	7 features, encoded as 1 if the participant was considered from this ethnic origin, 0 if not.
	Sex	”SEX”	Encoded as 0 if the participant was a woman, 1 if it was a man.
	Handedness	”HANDED”	3 features: ”RIGHT_HANDED”, ”LEFT_HANDED” and ”AMBIDEXTROUS” with 0 or 1 depending on the handedness of the participant.
Social	Years of education	”EDUCYRS”	Float corresponding to the n. of years of education of the participant.
Age	Age	”AGE”	Float corresponding to the age of the participant.
Parkinson’s features	Presence of tremor, rigidity, or postural instability at baseline	“DXTREMOR”, “DXRIGID”, “DXBRADY”, “DXPOSINS”	4 features encoded as 0 if the participants didn’t have this symptom, 1 if so. Bradykinesia was not mentioned in the paper but was used in the code, so we added this feature in our model.
	Dominant disease side	”DOMSIDE”	“DOMSIDE” feature column was used and split into 3 features: “DOMSIDE_LEFT”, “DOMSIDE_RIGHT” and “DOMSIDE_BOTH” with 0 or 1 depending on the dominant side of disease for the participant.
	Symptom duration and disease duration	”INFODT”, ”SXDT”, ”PDDXDT”	Symptom duration and disease duration: we respectively computed the number of days between the columns “INFODT” and ”SXDT” and between the columns “INFODT” and “PDDXDT”. Dates were first converted to “Month-Year” before computing the number of days between the two dates.
MoCA scores file	Baseline MoCA score	”MCATOT”	Integer value corresponding to the score of the participant. Missing values were replaced by the mean value across other participants. This information was not mentioned in code or paper.
GDS score file	Baseline GDS total score	”GDSSATIS”, ”GDSDROPD”, ”GDSEMPTY”, ”GDSBORED”, ”GDSGSPIR”, ”GDSAFRAD”, ”GDSHAPPY”, ”GDSHLPLS” ,”GDSHOME”, ”GDSMEMRY”, ”GDSALIVE”, ”GDSWRTLS”, ”GDSENRGY”, ”GDSHOPLS”, ”GDSBETER”	We computed the total score by summing all the columns containing “GDS” in the GDS short version study file. Missing values were replaced by the mean value across other participants.

Table 1. Demographics and clinical features set as input for the machine learning models. For baseline MDS-UPDRS scores included for prediction at 1 year, 2 years and 4 years, see section Outcome measurement.
