# Supplementary material for: Predicting Parkinson’s disease trajectory using clinical and functional MRI features: A reproduction and replication study
Source: PLoS One. 2025 Feb 21;20(2):e0317566. doi: 10.1371/journal.pone.0317566 (PMC11844873; doi:10.1371/journal.pone.0317566)
Supplement: S2 Table — (PDF) [file pone.0317566.s003.pdf]

Experiments	Cohort	Image preprocessing	ALFF/ReHo computation	Selection of input features	Workflow name
Reproduction	Closest-to-paper: n=82	Reproduction with AFNI segmentation	Z-score	All	Reproduction pipeline closest-to-original cohort
Variations of cohorts	Ours: n=102	Reproduction with AFNI segmentation	Z-score	All	Reproduction pipeline replication cohort
Variations of image pre-processing	Closest-to-paper: n=82	Reproduction with FSL segmentation	Z-score	All	Reproduction pipeline FSL segmentation
	Closest-to-paper: n=82	Reproduction without anatomic priors	Z-score	All	Reproduction pipeline no anatomic priors
	Closest-to-paper: n=82	fMRIprep pipeline	Z-score	All	fMRIprep pipeline
Variations of image feature processing	Closest-to-paper: n=82	Reproduction with AFNI segmentation	Not z-scored images	All	Reproduction pipeline no z-score
	Closest-to-paper: n=82	Reproduction with AFNI segmentation	ALFF instead of fALFF, Z-score	All	Reproduction pipeline ALFF
Variations of sets of input features	Closest-to-paper: n=82	Reproduction with AFNI segmentation	Z-score	No dominant disease side	Reproduction pipeline no domside
	Closest-to-paper: n=82	Reproduction with AFNI segmentation	Z-score	No baseline UPDRS	Reproduction pipeline no UPDRS
	Closest-to-paper: n=82	Reproduction with AFNI segmentation	Z-score	Only imaging features	Reproduction pipeline only imaging features
	Closest-to-paper: n=82	/	Z-score	No imaging features	No imaging features

Table 2. Terminology of the experiments in the current paper. Blue text denotes cohort or analysis variations that are closest to the original study, green text indicates variations due to unknown information, while red text indicates variations that are less similar to the original study.
