# Supplementary material for: Predicting Parkinson’s disease trajectory using clinical and functional MRI features: A reproduction and replication study
Source: PLoS One. 2025 Feb 21;20(2):e0317566. doi: 10.1371/journal.pone.0317566 (PMC11844873; doi:10.1371/journal.pone.0317566)
Supplement: Attachment — Submitted filename: answer-to-reviewers-egermani.pdf [file pone.0317566.s004.pdf]

Dear Editor-in-Chief, Co-editors, Reviewers,

We would like to thank the reviewers for their thoughtful comments. This document provides details on the updates performed in order to comply with the reviewer's comments. In the following, the reviewer's remarks appear in red **bold face**, our answers are in plain text and updates made to the manuscript are in *italic* and highlighted in different colors depending on the reviewer (this color-coded scheme is similar in the revised manuscript):

- Answers to Reviewer #1: *Cyan*
- Answers to Reviewer #2: *Yellow*
- Answers to Reviewer #3: *Pink*
- Answers to Reviewer #4: *Green*

**Reviewer #1: Review of PONE-D-24-21564: "Predicting Parkinson's disease trajectory using clinical and functional MRI features: a reproduction and replication study"**

**Kudos to the authors, for doing a great job of calling attention to the need for reproduction and replication studies. They provide examples of the challenges involved and suggest viable solutions. Reproduction and replication require detailed descriptions of procedures, which the authors elucidate and demonstrate by their example of detailed and meticulous work. The paper reads very well, despite numerous minor English language errors, which should be easy to correct by the English-language editor.**

We thank the reviewer for these comments.

**Minor items:**

**The applications of the terms reproduction and replication are a bit confusing as used in the paper, even though the authors define these terms on p 3, "Here, we define reproducibility as attempts made with the same methods and materials. Replicability, on the other hand, is tested with different but comparable materials or methods, assuming that the tested pipelines are all suitable to extract signal from the data." Rather than reproduction and replication, strictly speaking the authors demonstrate two flawed attempts at reproduction, one less flawed than the other, arguably approximating a true reproduction. The "reproduction" does not exactly reproduce the original work and the "replication" was not performed on (completely) different data. However, their approach is highly meaningful to help improve both reproduction and replication. Perhaps some more explanatory text could help to avoid confusion and point out that the authors' use of those terms is somewhat idiosyncratic.**

We agree with the reviewer that the terms reproduction and replication were sometimes confusingly used in the original manuscript. In this work, we made a two-step reproduction attempt: first, without contacting the authors, and second, with the help of the authors and with potentially original materials or methods. In the first step, we could not use the exact same methods as the original authors to analyze the materials, since we did not have access to the original image preprocessing pipeline. Thus, we built the closest-possible workflow (referred to as the default workflow in the paper) to try to reproduce the original one, but we agree that this default workflow is closer to a failed reproduction, and thus a replication, than to a reproduction.

Thus, we tried to modify the manuscript to avoid mentioning the term “reproduction” when mentioning our first attempt.

When we contacted the authors of the paper at the second attempt, they provided us with the derived data they used in the original study and we applied the same method as the authors. This attempt can indeed be defined as a true reproduction attempt. We made some corrections in the manuscript to clarify this and now refer to the first attempt as “Phase 1” and to the second attempt as “Phase 2”.

**P3. “Reproducibility and replicability of studies in clinical settings is of higher importance to improve the trustworthiness of new biomarkers and to facilitate their development.”--An excellent point. Perhaps this would be beyond the scope of the paper, but it might be worthwhile to also note that confounds such as residual noise (despite nuisance regressors) can affect the utility of the findings (e.g., would be no point in using a scanner as a measure of movement in the scanner if that is what ultimately affects the outcome of the analyses).**

We agree with the reviewer that reproducibility or replicability do not imply utility or validity. This point was also raised by Reviewer #2 and we would like to emphasize the fact that the goal of our study was to mainly explore the reproducibility and replicability of the findings, which are two important and necessary evaluation of the validity of the findings, but they are not sufficient. Other aspects would need to be explored to really assess the validity and utility of the biomarkers. As suggested by Reviewer #2 and in line with Reviewer #1’s comment, we added the following sentence to the manuscript:

*“Discussing the predictiveness of the extracted signals for the target outcomes found in the original study is out of the scope of this study. We focus on evaluating the impact of workflow variations in the prediction performance of the models.”*

**P3. “R2 greater than 0 and absolute difference between original and reproduction R2 less than 0.2.”--What led the authors to choose the value 0.2 for this test?**

We thank the reviewer for pointing out this lack of precision and we also noticed that this value was inconsistent across the paper, which is now fixed. This choice was made based on the original R2 values reported in the original study. In Nguyen et al., 2021, the lower R2 reported was obtained for prediction at Baseline time point using fALFF and was of 0.152. Since an R2 equal to 0 means that performance is similar to chance, we used the value of 0.15 as it represents the distance from 0 of the lowest R2 reported in the original study. The choice of this threshold means that 1) if we would have a difference higher than 0.15 compared to the original results, it would also mean that we would possibly obtain chance results and 2) a difference of 0.15 from chance level was considered as sufficiently high for the original authors to say that their model was making good predictions. We added a paragraph in the method section to clarify this:

“*We also compared the R2 values obtained with our different workflows with the original ones reported in [1]. We set a threshold of 0.15 to identify the workflows that were leading to important differences with the original ones. This threshold was chosen as it represents the lowest R2 reported across all experiments in the original study [1]. This means that this lowest reported R2 value was different from chance level with a threshold of 0.15. Thus, if we would have a difference higher than 0.15 compared to the original results, it would also mean that we would possibly obtain chance results. Moreover, this threshold was considered sufficiently high for the*

*original authors to say that the model was making good predictions. Thus, we kept this threshold to compare the performance of our models with the original ones.* ”

[1] Nguyen KP, Raval V, Treacher A, Mellema C, Yu FF, Pinho MC, et al. Predicting Parkinson’s disease trajectory using clinical and neuroimaging baseline measures. Parkinsonism & Related Disorders. 2021;85:44–51. doi:10.1016/j.parkreldis.2021.02.026

**P14. “However, we found higher mean disease durations in the replication cohort than in the original one at all time points, for instance at Baseline with (866.9 days ± 598.7 days) in replication vs (770 days ± 565 days) in original. This difference was not significant at threshold $p < 0.05$.” Please rethink the approach here and make adjustments. What does $p < 0.05$ reflect? Surely it does not reflect the probability of having such a large difference after randomly selecting subjects from the same population: The subject selection was not random at all. I think what the authors are trying to say is that in some cases there was nearly no difference in values (as expected for a true reproduction) and in another case the difference seemed meaningful. The authors can either leave this as an intuitive impression or perform some sort of test to illustrate the point, but a different test would be needed.**

We agree with Reviewer #1 that the statistical testing for comparing the different cohorts were not clearly described, except in the caption of Table 2, and that reporting p-values in this context does not make sense as populations are not sampled randomly. Thus, we removed any reference to p-values on that section (*Results - Cohort selection*) of the manuscript, and only compared cohorts by referring to features for which the difference between summary statistics was large.

**The figures are blurry. I have had the same problem, which in my case was fixed by changing a parameter in my Windows registry to set the DPI for the image to 300, after which I was able to export publishable figures from Microsoft Office programs.**

We thank the reviewer for this suggestion and re-uploaded our figures with the suggested modifications to make them less blurry.

**Reviewer #2: This study explores the replicability of clinical and imaging markers of Parkinson's disease diagnosis and progression prediction, specifically in the context of replicating the work of a previous study in terms of cohort selection, data preprocessing, feature selection/extraction, model selection, and feature importance pipelines. The write-up adequately reflects the undertaken methodical/analytical variations, similarities/differences with replicated work, and findings of the current work.**

We thank the reviewer for the comments. We answer to the following remarks and suggestion in the following:

**However, some serious study design flaws hinder the scientific utility and impact of this study as noted below. Foremost, while the write-up is well-detailed in mostly all parts, the structure and flow of the paper are lacking, and the results are not replicated on external data.**

We agree with the reviewer that the results were not replicated on external data. The main point of this paper was first to evaluate the reproducibility of the original study, and the main results of the study are the ability to retrieve similar results using the closest possible materials and methods. This reproduction process was challenging due to the update of the database used in the original study (i.e. PPMI) and the non-availability of some parts of the original code used by the authors (such as the image preprocessing pipeline). Thus, we had to make informed guesses to build the closest possible workflow and thus, also explored the robustness of the results to variations in the workflow (feature processing, cohort selection, etc.). We then found that results were sometimes different when varying the workflow. However, our goal was not to evaluate or to validate the biomarkers used in the original study, but only to explore their reproducibility and robustness to changes within the original workflow. Thus, we do not consider this goal necessitates a replication of the results on external data, as it is out of the scope of the paper.

**Specifically, the current flow of the manuscript in several parts is more like that of a rebuttal as compared to a collaborative effort with the authors of the replicated study. This suggests that the collaboration occurred only after a substantial effort had been made to individually replicate the results, thereby limiting the utility of that part of the current work.**

The reproduction and replication attempts presented in this paper were not meant to be a collaborative effort with the authors of the original study. This is mentioned at the beginning of the Materials and Methods section: "*Our study consisted of two steps: a first replication attempt without contacting the authors, using only publicly-shared resources available with the original paper, and a second replication attempt after contacting the authors, to obtain more accurate information on the original study*". The question of the utility of this part of the manuscript is answered in the following.

**Moreover, this limitation is unnecessarily amplified by differences in cohort use and even methodological variations between the previous and current studies. Even if it was a late collaboration, I don't see a reason why the exact data and pipeline used in previous work could not be shared between the collaborating teams, thereby eliminating the need to probe a majority of the considered differences and instead focus on enhancing the breadth and potentially the impact of the work (e.g., by exploring additional methods and most importantly replicate qualitative findings of putative biomarkers on additional datasets). As for the difference in the software versions, the previously used pipeline could have been containerized for replication.**

As explained above, this project was realized in two steps: a first reproduction attempt without contacting the authors and a second one in collaboration with the authors. In this second attempt, the authors of the paper provided us with the image preprocessing pipeline, the participants list of each time point and the derived data (i.e. fALFF and ReHo whole-brain maps) used in the original study. We first tried to apply the provided pipeline to our data from the closest-to-original cohort and we obtained results that were very different from the original ones, and from the ones that we obtained with our first attempt (i.e. with a reproduced pipeline). These differences were related to the fact that this pipeline was only containerized at the time of the actual study, and not at the time of the original one. Thus, updates were made on the authors' pipeline after the original study, and we were thus not able to retrieve the exact image processing pipeline used by the authors of the original study. This is why we chose to use derived data directly. This allowed us to validate the reproduction of the other parts of the workflow (i.e. clinical and demographic feature extraction, atlases and prediction models).

This is already described in the method section (see "Authors derivatives"). But, we emphasized this part to strengthen our motivation on using the derived data and not re-implementing the imaging pipeline:

*"While we could also have asked the authors for their original image processing pipeline, the retrieval of the exact version of the pipeline and software packages is challenging. The direct use of derived data allowed us to verify the reproduction of these steps and to get more information on the potential factors of variations in the results (e.g., suppressing differences in cohort selection and imaging processing, while retaining some potential differences in the version of scikit-learn)."*

**Lastly, while the central objective is to develop PD progression biomarkers, the qualitative findings of the current study indicate that the derived markers are not really replicable even for same/similar experimentation and within the same dataset. This replication failure (differences in biologically relevant information or the predictivity patterns of the input features) in this collaborative study questions the predictiveness of the extracted signals for the targeted outcomes and/or effectiveness/suitability of the undertaken training/inference approaches.**

We agree with the reviewer that our results question the predictiveness of the extracted signal and the suitability of fALFF and ReHo as PD progression biomarkers. We already discussed these findings in the Discussion section, in particular regarding the differences in predictive features and the importance of imaging features in the prediction: "*When fitting a machine learning model, similar performance can be achieved by different sets of features, which explains why feature importance values might be inconsistent across models*" and "*Some changes in the default workflow also led to lower performance, for instance at Year 1 and at Year 2 when removing Baseline MDS-UPDRS score or when using only imaging features. For these time points in particular, variations of the pre-processing pipeline (workflows B.), feature computation (workflows C.) and model choice and reporting (workflows E.) had little impact on the performance of the models compared to other time points. We speculate that imaging features were of low importance in the models prediction for these time points compared to other time points (Baseline and Year 4) for which variations on image computation (pre-processing or feature) had a larger impact.*". However, as stated before, our goal in this study was not to validate the biomarkers or not, but more to explore their robustness to workflow variations. Thus, we emphasize this in the Discussion by adding the following sentences:

“*Discussing the predictiveness of the extracted signals for the target outcomes found in the original study is out of the scope of this study. We focus on evaluating the impact of workflow variations in the prediction performance of the models.*”

**I hope the authors find my comments to be constructive for current/future work and wish them success in their research.**

**Reviewer #3: Elodi et al. have replicated and reproduced the study for predicting Parkinsons disease trajectory based on previous study and available information in PPMI database . They have done comprehensive study and have address the problem in reproducing study on predication of parkinson trajectory.**

**I find the manuscript organised and well written with clear goals. Results are well described and is supported by data. I recommend it for publication.**

We thank the reviewer for the kind comments.

**Reviewer #4: The authors performed an interesting study that tackles an important topic in the attempt to develop neuroimaging biomarkers for clinical implementation in Parkinson's disease: the robustness of findings. Features are derived from functional MRI and a machine learning model is used to predict disease severity. The methods are thorough and the conclusions are insightful. I do have concerns, however, which largely revolve around the contribution of some of the original authors as co-authors. Their involvement and openness is crucial to performing this study well, but inherently creates a conflict of interest that needs to be addressed more explicitly. Firstly, I urge the authors to add a statement on how neutrality is safeguarded. Secondly, to ensure that the manuscript resonates with all readers and upholds the highest standards of impartiality, I would kindly suggest revisiting the sections where the narrative might seem biased (e.g. remove intensifier words). Further, it is important to clearly define when model performance and feature importance are regarded as different from the original. To do this, it needs to be specified why specific thresholds were used to determine difference between models. Finally, the readability of the manuscript can be improved upon, as a lot of analyses are performed and the reader may get lost in the methods and results. I have provided more details about these and other comments in a point-by-point fashion below.**

We thank the reviewer for the comments and we answer each of these points below.

**Detailed points**

**Abstract**

**1. "Several neuroimaging biomarkers have been studied recently, but these are susceptible to several sources of variability."**

**I think it would help to more concretely define the type of variability of interest for this study. As other types of variability exist as well, but are not covered in this manuscript.**

We added specifications in the abstract "*... to several sources of variability related for instance to cohort selection or image analysis. In this context, an evaluation of the robustness of such biomarkers to variations in the data processing workflow is essential.*".

**2. I suggest to briefly define replication and reproduction in the abstract.**

This is already done in the abstract, but we emphasized these definitions: "*Here, we attempt to reproduce (re-implementing the experiments with the same data and same method) and replicate (with different data and/or method)...*".

**3. "The success of the reproduction was assessed using different criteria."**

**This phrasing is too vague and I suggest to rephrase it.**

We rephrased this sentence according to the reviewer's suggestion:

"*Different criteria were used to evaluate the reproduction and compare the reproduced results with the original ones*."

**4. Only the replication results are mentioned and not the reproduction results. To accurately reflect the contests of the manuscript, those results should be summarized here as well.**

We agree with the reviewer and added a sentence in the abstract: "Notably, we obtained significantly better than chance performance using the analysis pipeline closest to that in the original study ($R2 > 0$). *Moreover, using derived data provided by the authors of the original study, we were able to make an exact reproduction and managed to obtain results that were close to the original ones.* "

**Introduction**

**5. The first paragraph mentions "disease subtypes" on various occasions, but this is not studied in the current manuscript. It may be more clear to only mention stages, severity or progression, as that aligns more closely with the manuscript.**

We agree with the reviewer and removed any mention of disease subtypes in the introduction.

**6. "While disease phenotypes are heterogeneous, neuronal dysfunction patterns were shown to be highly replicable between patients."**
**I would like to see the latter statement explained more in depth. It also seems to be phrased a bit too strongly, also considering the explained variance in the final models of this study. These patterns can still be important, but I think more contextualization is important.**

We agree with reviewer and added the following text:

"*While disease phenotypes are heterogeneous, neuronal dysfunction patterns were shown to be highly replicable between patients. In [6], authors showed that while the location of the dysfunction within brain networks might vary between individuals, the progression of this dysfunction over time, associated with the progression of the disease itself, was shown to be highly similar between individuals.*"

**7. Why may the ALFF and ReHo measures be particularly relevant? This needs to be apparent for the reader without requiring them to read the original manuscript.**

We added the following sentence in the Introduction to explain the choice of ALFF and ReHo:

"*ReHo quantifies the connectivity between a voxel and its nearest neighboring voxels and was shown to be affected by neurodegenerative diseases [12]. ALFF and its normalized form, fractional ALFF (fALFF), measure the power of the low frequency signals at rest, which mostly consists in spontaneous neuronal activity [13]. In previous studies, specific regional values of these two measures (e.g. ReHo in the putamen and cerebellum, and fALFF in the right cerebellum) were found to be correlated positively or negatively with MDS-UPDRS scores. These findings have been attributed to the role of several brain networks involving these regions in motor function.*"

**8. "This suggests that a single pipeline evaluation is not sufficient to obtain robust results, though the reliability of results may be increased when studying their distributions across perturbations."**
**The second part of this sentence may be a bit difficult to follow for non-expert readers. I suggest to give a little more context**

We modified the sentence according to the reviewer's comment:

"*This suggests that a single pipeline evaluation is not sufficient to obtain robust results. A poor robustness of the results would question their reliability, since significant results might have been obtained by chance and might actually be false positive findings [19]. This robustness can be assessed by studying the distribution of results across perturbations of the workflow.* "

[19] John P. A. Ioannidis. Why Most Discovered True Associations Are Inflated:. Epidemiology, 19(5):640–648, 2008. ISSN 1044-3983. doi: 10.1097/EDE.0b013e31 818131e7.

**9. "Note that comparable is ambiguous, but defined further in this case in Method Section."**
**It may be helpful to at least briefly summarize the definition here. Also, the second part of the sentence does not read very well. I suggest rephrasing it slightly.**

We rephrased this sentence according to the reviewer's comment:

"*Note that the term comparable is ambiguous, but we define its use in the context of this study in the Method Section.*"

**10. "Replicability experiments have shown different degrees of variability between findings obtained with different analytic conditions. These studies are usually done using healthy populations and in general research practice (as opposed to clinical research), as in [16]. For clinically-oriented research, however, the topic remains understudied."**
**The difference between clinically-oriented research and general research practice needs to be defined more clearly.**

We agree with the reviewer and chose to remove the term "*general research practice*". We also added a clarification: "*For clinically-oriented research, i.e. using patient populations,...*"

**11. "Reproducibility and replicability of studies in clinical settings is of higher importance to improve the trustworthiness of new biomarkers and to facilitate their development"**
**Until now the introduction mostly covers the robustness and variability of biomarkers, which clearly links to the trustworthiness of the findings. However, I suggest to provide more details on how this facilitates the development of them.**

We believe that the trustworthiness of the findings is directly related to the development and application in clinical practice of biomarkers. However, we agree with the reviewer that the sentence was not clear and modified it in the revised manuscript:

"*Reproducibility and replicability of studies in clinical settings is of higher importance to improve the trustworthiness of new biomarkers, which is an important factor that would facilitate their development and application in clinical practice.* "

**12. "… a clinically-oriented research …"**
**This seems grammatically incorrect. I suggest to change "research" to "study".**

We made the modification according to the reviewer's suggestion.

**13. The involvement of authors of the original paper (e.g. KPN and AAM) is crucial to make accurate comparisons to that work. Their openness to share resources, as well as being involved in the study despite some slightly conflicting results being reported is commendable. Unfortunately, even with the best intentions, their direct involvement as co-authors does make it difficult to judge the independence of the statements made in the manuscript. For transparency, I think it is important to add a statement mentioning how this was handled.**

We agree with the reviewer that the involvement of authors of the original paper can create some bias. In the revised manuscript, we tried to emphasize the separation of our work into two phases: a first phase (Phase 1 in the revised manuscript), without contacting the authors, which relates more to the definition of a replication since we did not have access to the same materials and methods, and a second phase in collaboration with the authors (KPN and AAM) in which they shared supplementary materials that allowed us to perform a proper reproduction. In line with the first Minor Item of Reviewer #1, we emphasized the separation of these two phases and made the results of the different phases more distinguishable in the Tables and Figures captions.

*"Our study consisted of two steps:*

- *Phase 1: a first reproduction attempt without contacting the authors, using only publicly-shared resources available with the original paper.*
- *Phase 2: a second reproduction attempt after contacting the authors, to obtain more accurate information on the original study."*

**14. The abstract mentions that this study is part of a larger effort to replicate imaging biomarkers in PD. This should be mentioned in the introduction as well. On top of that, given the context of this broader effort, I suggest to specify what sets this specific study apart.**

We added a sentence at the end of the Introduction section to explain this:

*"This study is part of a larger effort to explore the reproducibility and robustness of PD biomarkers extracted from neuroimaging data, but this study is the first to explore the robustness of fMRI related biomarkers of PD."*

In the future, a paper summarizing the conclusions of the different reproducibility experiments of this effort will be published and more details will be provided regarding the specificities of this study compared to the other ones.

**Methods**

**15. The terms reproduction and replication have been well defined in the introduction. However, throughout the manuscript they seem to not have been used fully consistently. For instance, the first paragraph of the methods contains the phrase "This two-step reproduction was meant…". However, reproduction here refers to both reproduction and replication. I recommend thoroughly checking this throughout the manuscript to avoid confusion.**

This comment aligns with Reviewer #1 and we agree with the Reviewers that the terms were inconsistent throughout the manuscript. Thus, we made some modifications to clarify this and to avoid using the term reproduction.

**16. The choice to start with a replication step without contacting the authors should be motivated in more detail in the introduction. Currently it is not made clear enough as to why this would be an important first step.**

In order to emphasize this, we added a sentence in the Introduction section that summarizes our statements regarding the two steps reproduction (which is developed in more detail in the Method section).

"*Our main interests were to assess the difficulties and challenges of reproducing fMRI research experiments, thus we first tried to reproduce the study without contacting the authors to assess the importance of publicly-shared resources and description given in the paper. After that, we contacted the authors to better understand the failure of our initial reproduction.*"

**17. Relatedly, for workflows as well as cohorts, it was hard to keep track which related to replication and which to reproduction. This needs to be made more clear. A suggestion would be to consistently use the words "replication" and "reproduction" when naming the workflows and cohorts.**

This comment also relates to Item 15 and to Reviewer #1's comment. We made modifications to consistently use the different terms and avoid any misunderstanding.

**18. The "default workflow" was defined as the workflow that was "most likely" according to the code shared along with the paper. It is not fully clear wat "most likely" means here. This is explained more thoroughly in the paragraphs below, but it may help to briefly inform the reader that details will be given below.**

We added a sentence according to the reviewer's suggestion:

"*More details will be given below regarding the choices that we made to build this pipeline.*"

**19. The scanning acquisition parameters need to be included.**

The scanning acquisition parameters are already included in the Cohort selection subsection since we used these parameters to filter the PPMI database: "*(field strength = 3T, scanner manufacturer = Siemens, pulse sequence = 2D EPI, TR = 2400ms, TE = 25ms)*".

**20. The authors chose to filter the database and keep only the participants for which the visit/archive date was before January 1st, 2020. I am wondering why a date was used an not use the number of participants for this? Selecting a certain date feels more arbitrary.**

During the Phase 1 of the project, we did not have any contact with the authors and thus, we had to reproduce as closely as possible the filtering of the PPMI database that was performed at the time of the original study. To do so, other filtering approaches could have been used such as limiting the number of participants or not including any date. But, here, we chose to use a date to

filter the database to mimic as closely as possible the original conditions to try to reproduce the filtering method. However, we showed in the paper that this cohort filtering was not reproducible due to updates of the database and absence of version controlling of the PPMI database (we refer the Reviewer to the Discussion for more details on this part). We added a sentence in the paper to clarify our choice:

*"Note that the choice of this date was made to reproduce as closely as possible the condition of the original database filtering, but other filters could have been used."*

**21. A few variations of the original pipeline were tested to see how these would affect the results. It is not fully clear, however, why these particular variations were chosen. I would like to ask the authors to elaborate on this.**

We added a sentence at the beginning of the section "Variations of the default workflow" to better explain the choice of these particular variations:

"*As mentioned in the previous section, we had to make guesses to build the pipeline for the default workflow. For some of these guesses, other valid alternatives would have been possible. In particular, for the extraction of WM and CSF, which could have been made with another software package and/or method.*"

Also, regarding the section "Other pipelines variations", the choice of using fMRIprep as a comparison was to make comparison with a more distant pipeline, as variations of the default workflow remain close to the default one (and to the original one). We added a sentence about this:

"a robust pre-processing pipeline that requires minimal user input*, and which implements pre-processing steps that are different from the ones used in the default workflow and its variations. This allowed us to see how impactful the changes in image pre-processing pipelines could be in this study.* "

**22. "We note that for the second step of the reproduction experiment, the authors of [1] have supplied us with all derived maps."**
**Here, it is not completely obvious what "second step" refers to.**

We clarified this in the text:

"We note that for the second step of the experiment *(i.e. after a reproduction attempt using only publicly-available materials, by contacting the authors)*"

**23. The discussion about the atlases in the "imaging features computation" section should be in a distinct subsection.**

We agree with the reviewer and separated this section into two subsections:

- 1) Whole-brain maps computation
- 2) Regional features extraction

**24. "… they had downloaded, through they did factor prominently into their results, in**

**order to understand better the relevance of the database update."**
**The final part of this sentence does not seem grammatically correct. There is also a typo here: through --> though**

We thank the reviewer for pointing out this mistake and we made the appropriate corrections in the manuscript.

**25. In the section on "Evaluation metrics", I suggest to also explain what the RMSE represents, just like was done for R2.**

We modified this paragraph according to the reviewer's suggestion:

*"As in the original paper, performance metrics included the coefficient of determination (R2), which represents the percentage of variance explained by the model, and the root mean squared error (RMSE), which represents the root mean squared difference between true and predicted values, as implemented in scikit-learn."*

**26. Null performance was tested using the ElasticNet and Schaefer atlas, but it is not explained why these were chosen. Please elaborate on this.**

Indeed, we did not explain this choice in the original manuscript. We added a sentence to explain in the revised version:

"*This model and parcellation scheme were chosen as these were the most identified as best performing models across all time points and features in the original study.*"

**27. Was the evaluation of the models' ability to classify high versus low severity patients also evaluated in the original study? This is not fully clear to me from the current text.**

Yes, this evaluation was also performed in the original study to evaluate the models using metrics such as accuracy. We added this precision in the revised manuscript.

"*To evaluate the models' ability to classify high versus low severity participants, as it was performed in the original study [1],...*"

[1] Nguyen KP, Raval V, Treacher A, Mellema C, Yu FF, Pinho MC, et al. Predicting Parkinson's disease trajectory using clinical and neuroimaging baseline measures. Parkinsonism & Related Disorders. 2021;85:44–51. doi:10.1016/j.parkreldis.2021.02.026

**28. It needs to be defined when model performance will be regarded as different from the original and when they will be regarded as similar. The same is true for feature importance.**

This comment is in line with Minor Item at P3 of Reviewer #1. We added details on the manuscript to explain how the threshold of 0.15 was chosen to define model performance that was different from the original.

Regarding feature importance, we only compared the list of most important features obtained when running the corresponding authors code with the features reported in the original study. The features that were different between our model and the original study were noted with a star

symbol in Figures 5 and 6. This information is already present in the caption of both figures.

**Results**

**29. Throughout the results section, statistical parameters either seem to be missing or simply limits being reported (e.g. <0.05). These parameters need to be reported more comprehensively and with exact values.**

We understand the reviewer's point and this comment is in line with Minor Item P14 of Reviewer #1: reporting p-values in this context does not make sense as populations are not sampled randomly. Thus, we removed any reference to p-values on that section (*Results - Cohort selection*) of the manuscript, and only compared cohorts by referring to features for which the difference between summary statistics was large.

**30. When differences were observed between cohorts, I suggest to specify the values of each cohort, similarly to what was done for disease duration in paragraph 3.**

We specified the values for which cohorts showed significant differences in Paragraph 3 as suggested by the reviewer.

"*Compared to the original cohort, our replication cohort showed similar demographics characteristics at each time point, except at Year 4 where our replication cohort showed a significantly higher age on average than in the original cohort (66.2 ± 10.1 years compared to 59.5 ± 11.0). Regarding clinical variables, mean MoCA score, GDS total score and Hoehn-Yahr stage were similar between the two cohorts at all time points. However, we found higher mean disease durations in the replication cohort than in the original one at all time points, for instance at Baseline with (866.9 days ± 598.7 days) in replication vs (770 days ± 565 days) in original. We also observed lower baseline mean MDS-UPDRS scores in the replication cohort for all time points except Baseline, in particular for Year 2 with a mean baseline score of 35.2 ± 16.1 compared to 40.2 ± 18.2 in the original cohort. For the two time points Year 2 and Year 4 where we mostly found differences, even if mean Baseline scores in the replication cohort differed from the original ones, mean MDS-UPDRS scores at prediction time point were more similar to the original one. At Year 4, however, we also found a higher mean MDS-UPDRS score at prediction time point (30.7 ± 13.9) than in the original cohort.*

*The closest-to-original cohort exhibited almost the same characteristics as the original one at Baseline. For subsequent time points, we found some differences, in particular at Year 2 and at Year 4: participants were older in the closest-to-original cohort than in the original study at Year 4 (63.8 ± 11.0 in the closest to original cohort compared to 62.1 ± 9.8 in the original), Baseline mean MDS-UPDRS score was lower for Year 2 (40.2 ± 18.2 in original, 35.2 ± 16.1 in closest-to-original) and Year 4 (34.9 ± 15.7 in original, 26.1 ± 11.4 in closest-to-original) and mean MDS-UPDRS score at prediction time point was similar to the original cohort except at Year 4.*"

**31. "Most participants of the study showed high movement parameters. Indeed, out of 102, 80 showed at least one time point with a frame-wise displacement superior to 0.5mm."**

**What was the mean frame-wise displacement?**

We added a Supplementary Table with the mean frame-wise displacement across time points for each of the 102 participants. We also report the mean frame-wise displacement averaged across all time points and all participants in the revised manuscript:

*"The mean frame-wise displacements across all time points for each participant are reported in Supplementary Tables. The mean frame-wise displacement across time points and participants was of 0.258."*

**32. Sometimes the R2 is reported as fraction and sometimes as percentage. This needs to be the same throughout the results.**

We understand the comment of the reviewer. We removed any reporting that used percentages in the manuscript and replaced those with comparison or difference between values.

**33. The results section contains too much discussion and interpretation that are repeated in the discussion. Readability of the manuscript would be improved if this section is written more concisely.**

We agreed with the reviewer and removed some sentences from the Results section that were repeated in the Discussion. For instance, we **removed** the following sentences from the Results section to avoid repetitions:

"*We can also suppose that the variations observed between the performance of the default workflow and the original results are related to imaging features (pre-processing or feature computation) or differences between cohorts.*"

We also moved some sentences to the Discussion:

"*These differences for the Year 1, Year 2, and Year 4 cohorts could be related to the evolution of the PPMI database in which sessions were added and removed since the authors queried it for the original study.* "

"*This is entirely plausible for multivariate machine learning models, and does not preclude the other set of features from not also being useful (e.g. if default gets 0.717, it could be that the features from original are still informative of outcome)*"

"*This highlights the importance of model selection and performance reporting, which were also featured prominently in [1]. Some models may have not been optimally tuned, and all models do not have equal capability due to their different functioning, leading to lower performance. These low performance obtained with some models do not put into question the other results, as these have been validated on an external dataset by [1].*"

I hope that these changes will help to improve the readability of the manuscript.

**34. "In Fig 4, we can see that using authors derivatives and thus, the original cohort, we achieve performance that are very close to the original ones, except at Year 4. This informs us on the quality of the reproduction of the clinical and demographic features selection, but also on the machine learning models training and selection"**

**The observation that the performance is not the same for year 4 should be addressed. If this is not close to the original values, then something must be different.**

We agree with the reviewer that this difference should be assessed and discuss it in Item 38 to avoid including too much discussion in the results.

**35. In the “robustness to workflow variations” section, “models performance” is grammatically incorrect.**
**36. Typo: “…values with for fALFF..” --> for**

We thank the reviewer for pointing out these two errors and made the appropriate corrections.

**37. “Feature importance showed relatively few overlap between the ones obtained using our models and those reported in the original study, especially for imaging features, at all time points”**
**This is an important point that needs to be covered more extensively in the discussion, as it calls the reliability of the imaging measures into question.**

With changes made to answer to Item 33, we added more details to this in the Discussion:

*“In addition, using our default workflow, we found feature importance values that differed ---for some predictions--- from the ones found by the authors. This is entirely plausible for multivariate machine learning models, and does not preclude the other set of features from not also being useful (e.g. if default gets 0.717, it could be that the features from original are still informative of outcome). [...] When fitting a machine learning model, similar performance can be achieved by different sets of features, which explains why feature importance values might be inconsistent across models.”*

**Discussion**
**38. “When training the models using the derived data computed by the authors at the time of the original study (fALFF and ReHo whole-brain maps), we found very close performance to the original ones, except at Year 4 with fALFF, where the default worklow found even higher predictability. This confirms the quality of the reproduction for the clinical and demographics feature selection and for the machine learning part.”**
**A higher predictability at Year 4 with fALFF still is a different performance. This was a recurrent statement throughout the discussion that needs to be addressed accordingly.**

We agree with the reviewer that even with a closer reproduction using original derived data provided by the authors, we managed to obtain a higher predictability at Year 4 with fALFF. The goal of this reproduction in Phase 2 was to assess the reproduction of the prediction and feature extraction parts of the workflow. However, it remains some slight differences between our reproduction and the original study that can explain the higher predictability observed:

- Updates of the PPMI database: imaging features were obtained from the original derived data, but clinical and demographic features were extracted from the actual PPMI database, which was updated since the study.
- Cross-validation scheme and selection of hyperparameters which is a complex step to reproduce, as explained in the paper.

- Differences in scikit-learn versions,
- etc.

We added a sentence in the revised manuscript to discuss this:

"*Note that, since we used clinical and demographic features available in the PPMI database at the time of this study when training models with derived data, the difference of selected session in each cohort (in particular at Year 4) might also explain the higher predictability observed at that time point during Phase 2 when traning models with derived data. Other factors such as differences in scikit-learn version or differences in cross-validation schemes and hyperparameter selections might have impacted the results of Phase 2 experiments.*"

**39. In that same sentence there is a typo: worklow --> workflow**
**40. "When using a different cohort with distinctions in the distribution of the most important feature (MDS-UPDRS score at Baseline) of the Year 1 model"**
**Given all the workflow and cohorts that the reader needs to track, I suggest being more specific than "a different cohort" and use names of cohorts consistently.**

We made the appropriate correction for the two previous points in the revised manuscript.

"*When using the replication cohort in which there are differences in the distribution of the most important feature…*"

**41. "It would be interesting to assess the interaction of variations in both cohorts, imaging features and input features sets to see if the robustness to analytical variations was also present using the replication cohorts and when increasing the importance of image features in the prediction."**
**What answers could this give exactly? And, if interesting, why not do it?**

This would be useful to explore in more depth the robustness of the findings. Indeed, even if it is out of the scope of the study, our findings question the predictiveness of imaging features fALFF and ReHo in PD progression. These further experiments would allow us to see if the prediction performance is also robust to changes in imaging features in another cohort (here, the replication one), which is larger and thus, potentially leading to a more efficient training. However, this is out of the scope of the paper, but would be useful to validate or not the predictive power of the features used in this study.

**42. "…we first attempted to query the PPMI database using the information available in the paper and the code shared by the authors."**
**I believe this refers to the publicly available code, but since the original authors were included later and shared resources at that point as well this may be confusing. I suggest clarifying this.**

We clarified this in the revised manuscript:

"*...the information available in the paper and the code publicly available at the start of the reproduction (i.e. without contacting the authors).*"

**43. In the discussion it was noted that five participants had a different visit in the closest-to-original cohort, but the results only mentions four. Please check this.**

This was a mistake, and indeed, only four participants had a different visit in the closest-to-original cohort. We corrected this in the revised manuscript.

**44. The discussion describes that select software could help with replicability, but it is still unclear which specific software tackles what specific challenges. I would like to see more detail on this.**

We believe the reviewer refers to the part of the Discussion on the use of containerized software packages. We added a reference in the revised manuscript to a recent review on software tools that help to study and improve reproducibility:

*"For more details on the benefits of such software containerization, we refer the readers to [60] in which authors explain how using these particular frameworks can help reproducibility."*

[60] Botvinik-Nezer R, Wager TD. Reproducibility in Neuroimaging Analysis: Challenges and Solutions. Biological Psychiatry: Cognitive Neuroscience and Neuroimaging. 2023;8(8):780–788. doi:10.1016/j.bpsc.2022.12.006.

**45. Relatedly, it would be helpful to add a box/figure with very specific point-by-point recommendations for future studies. I think this could facilitate adoption of your recommendations.**

In accordance with the reviewer comments, we added a box at the end of the discussion to highlight our main recommendations to improve the reproducibility of future studies.

**46. The discussion contains several intensifiers that add subjective emphasis (e.g. "slightly" or "very") when describing differences to the original study. These need to be used more carefully.**

We agree with the reviewer and tried to remove intensifier words in this Section in the revised manuscript.

**47. Relatedly, the last sentence notes that "reproducing existing papers remains extremely challenging", whilst the discussion often notes how the reproduced results were close to the original. This discrepancy is confusing and should be addressed.**

In this sentence, we mostly refer to the reproduction of the exact workflow used in papers. We clarified this in the sentence: "*reproducing the experiments made in existing papers*".

**48. Some additional limitations need to be mentioned, such as those mentioned in the original manuscript.**

We added the original limitations in the revised manuscript as suggested by the reviewer.

*"Beyond the limitations related to the challenges of reproducibility, all limitations identified by the authors of [1], including bias of the PPMI cohort towards Caucasian, the use of small sample sizes in particular for prediction at Year 4 and the impact of medication on MDS-UPDRS scores, are also applicable for our study and are further discussed in the original paper [1].*"

**Figures**

**49. Figure 1 is referenced as "Figure 1" on one occasion, whereas all other references to the figures use "Fig" in the main text. I suggest to align this.**

This point was also noted by the Editor, and we made modifications to align all mentions to figures with "Fig" according to the Editor's guidelines.

**50. Figure 1. It would be helpful if here the authors could visualize which workflows count as "replication" and which as "reproduction".**

We agree with the reviewer and added a precision in the caption of this figure:

"*The different replication workflows are represented in dashed lines: all steps different from the variation follow the default workflow and each workflow corresponds to one variation from the default one. The reproduction workflow obtained with derived data provided by authors of [1] is represented with a point-style line.* "

**51. Figure 3. The binning of the replication cohort at year 1 seem different from the closest-to-original and original cohorts. This may need to be adjusted for accurate comparisons.**

The binning is actually similar between the two graphs and set according to the original code publicly-available from Nguyen et al., 2021.

**52. Figure 3. Why were Year 2 and 4 not reported?**

In the original study of Nguyen et al., 2021, only distribution of MDS-UPDRS scores for cohorts at time points Baseline and Year 1 were reported. Thus, we are not able to compare the distribution of scores of our cohorts with the original one for Year 2 and 4, which is why we did not include them in the Figure.

**53. Figure 6. I suggest to use the same coloring scheme for easier visual comparisons of the two atlases.**

We agree with the reviewer on his comment, however, due to the difference in terms of organization, order and labeling of the different regions in the two atlases, it is not possible to apply the same coloring scheme.
