# Supplementary material for: Predicting Parkinson’s disease trajectory using clinical and functional MRI features: A reproduction and replication study
Source: PLoS One. 2025 Feb 21;20(2):e0317566. doi: 10.1371/journal.pone.0317566 (PMC11844873; doi:10.1371/journal.pone.0317566)
Supplement: Attachment — Submitted filename: answer-reviewers-egermani.pdf [file pone.0317566.s005.pdf]

Dear Editors and Reviewers,

We would like to thank the reviewers for their thoughtful comments, and for recognizing our efforts. This document provides details on the updates performed in order to comply with the reviewer's comments. In the following, the reviewer's remarks appear in red **bold face**, our answers are in plain text and updates made to the manuscript are in *italic* and highlighted in different colors depending on the reviewer (this color-coded scheme is similar in the revised manuscript):

- Answers to Reviewer #1: *Cyan*
- Answers to Reviewer #4: *Green*

Reviewer #1: Review of PONE-D-24-21564_r1: "Predicting Parkinson's disease trajectory using clinical and functional MRI features: a reproduction and replication study"

The authors have done a fine job of meticulously addressing reviewer comments.

We thank the reviewer for their comments.

However, two of my comments have not been fully addressed:

The language concerning the terms reproduction and replication has been much improved and far less confusing, but there are still some points of potential confusion. The authors seem to contradict themselves at times. For example, in the abstract "Here, we attempt to reproduce (re-implementing the experiments with the same data, same method) …" defines a reproduction as involving not only the same methods, but also the same DATA. In the abstract we also find "Moreover, using derived data provided by the authors of the original study, we were able to make an EXACT reproduction and managed to obtain results that were close to the original ones." This begs the question, if the same data and same methods were used, why aren't the results the same? I don't find this question answered.

From lines 601-608 we find "The closest-to-original cohort exhibited almost the same characteristics as the original one at Baseline. For subsequent time points, we found some differences, in particular at Year 2 and at Year 4: participants were older in the closest-to-original cohort than in the original study at Year 4 (63.8 ± 11.0 in the closest to original cohort compared to 62.1 ± 9.8 in the original), Baseline mean MDS-UPDRS score was lower for Year 2 (40.2 ± 18.2 in original, 35.2 ± 16.1 in closest-to-original) and Year 4 (34.9 ± 15.7 in original, 26.1 ± 11.4 in closest-to-original) and mean MDS-UPDRS score at prediction time point was similar to the original cohort except at Year 4." And on lines 902-905 "Even with the original participant identifiers and visit list at Baseline, we could not retrieve the same Baseline cohort in the PPMI database. Our closest-to-original cohort included the 82 original participants, but for 4 of them, a different visit than the original one was used." Are the authors actually dealing with the EXACT same data in their reproduction? Perhaps the authors could avoid

confusion by qualifying that the data are nearly exactly the same and/or that the reproduction was attempted rather than an EXACT reproduction.

We thank the reviewer for pointing us to these confusions. We removed any mention of "exact reproduction" and clarified that our use of "same data" refers to using the same database of origin (i.e. PPMI). In Phase 2, two reproduction attempts were performed:

- A **full reproduction attempt,** in which we tried to reimplement all the experiments, including the cohort selection and data collection step from the same database using the original authors' (KPN and AAM) participants and session list. This corresponds to our "closest-to-original" cohort.
- A **partial reproduction attempt**, where we tried to reimplement the experiments using the derived data provided by the original authors, bypassing the data collection step to focus on reproducing the machine learning and feature extraction methods.

To avoid confusion, we distinguished the **full reproduction** attempt in which data are obtained from the same database using the same data collection method, and the **partial reproduction** attempt in which we used the same derived data as in the original study. We made the following changes in the manuscript:

Abstract:

- "We attempt to reproduce (*reimplementing the experiments with the same methods, including data collection from the same database*)..."
- *"In addition, we performed a partial reproduction using derived data provided by the authors of the original study, and we obtained results that were close to the original ones."*

Introduction:

- "Here, we define reproducibility as attempts made with the same methods and materials. *The aforementioned method can include a data collection step, and in that case, we consider that the term same materials corresponds to the original database where data collection is performed."*

Method:

- *"In Phase 2, we performed two different reproduction attempts: a full reproduction, including the cohort selection and data preprocessing steps, and a partial reproduction, using the derived data provided by the original authors."*
- "In Phase 1, we attempted to reproduce *the cohort selection process*…"
- "*This "closest-to-original" cohort corresponds to the one obtained in Phase 2 with a full reproduction attempt of the experiments: the same data collection strategy as the original authors and the list of participants and sessions of the different cohorts provided by the original authors. Note that even using the same data collection strategy and the participants list, retrieving the same data was not possible due to the changes in the PPMI database mentioned above.*"

Results:

- "The closest-to-original cohort, *obtained using the same cohort selection method as in the original study*, exhibited almost the same characteristics as the original one at Baseline."

To answer the reviewer’s question about the differences in the results obtained using the partial reproduction (same derived data and same methods), we added the following sentence:

- “*This reproduction will be referred to as “partial reproduction”: we used the derived data provided by the authors and thus, did not attempt to reproduce the cohort selection and pre-processing steps. In that case, we consider that the data are the same as the ones used in the original study. Thus, any divergence between our results and the original ones would be explained by potential differences in the version of scikit-learn, the non-determinism of algorithms, or differences in computer environments.*”

This question is also answered in the Discussion part, in which we emphasized on the potential reason for these different results:
“This confirms the quality of the workflow reproduction for the clinical and demographics feature selection and for the machine learning part. *Note that, only derived rs-fMRI data were provided by the authors, the clinical and demographic features used in this partial reproduction attempt are the ones available in the PPMI database at the time of this study. Thus, potential updates in the PPMI database and on the features might explain the higher predictability observed at that time point during this partial reproduction attempt. Other factors such as differences in scikit-learn version or differences in cross-validation schemes and hyperparameter selections might have impacted the results of the partial reproduction experiments.*”

The figures are still blurry, but this issue can wait until final preparation for publication.

We are very sorry for this inconvenience and are aware of this issue. This is due to the conversion of figures from PNG or PDF to TIFF format, which is required for submission at PLOS ONE. We will work on this issue before final preparation for publication.

Reviewer #4: I would like to thank the authors for addressing all my comments. All my concerns have been resolved. I have one final comment regarding the removal of p-values, which I agree with as the samples have not been gathered randomly and independently. However, a definition of when values are considered "different" is missing and I suggest specifying this.

We thank the reviewer for their comments. We added a definition of the criteria we used to determine whether values were considered different or not:

"*To evaluate the differences between the original cohort and our two cohorts, we determined whether the values obtained for our cohorts fell within a ±10% range of the original value.*"

Values that did not fulfill these criteria are in bold in Table 2 of the revised manuscript.
